# A first insight into the genome of *Prototheca wickerhamii*, a major causative agent of human protothecosis

**DOI:** 10.1186/s12864-021-07491-8

**Published:** 2021-03-09

**Authors:** Zofia Bakuła, Paweł Siedlecki, Robert Gromadka, Jan Gawor, Agnieszka Gromadka, Jan J. Pomorski, Hanna Panagiotopoulou, Tomasz Jagielski

**Affiliations:** 1 Department of Medical Microbiology, Institute of Microbiology, Faculty of Biology, University of Warsaw, I. Miecznikowa 1, 02-096 Warsaw, Poland; 2 Department of Systems Biology, University of Warsaw, I. Miecznikowa 1, 02-096 Warsaw, Poland; 3 Department of Bioinformatics, Institute of Biochemistry and Biophysics, Polish Academy of Sciences, A. Pawińskiego 5a, 02-106 Warsaw, Poland; 4 DNA Sequencing and Synthesis Facility, Institute of Biochemistry and Biophysics, Polish Academy of Sciences, A. Pawińskiego 5a, 02-106 Warsaw, Poland; 5 Museum and Institute of Zoology, Polish Academy of Sciences, Wilcza 64, 00-679 Warsaw, Poland

**Keywords:** Alga, *Prototheca wickerhamii*, Protothecosis, Virulence, Whole genome sequencing

## Abstract

**Background:**

Colourless microalgae of the *Prototheca* genus are the only known plants that have consistently been implicated in a range of clinically relevant opportunistic infections in both animals and humans. The *Prototheca* algae are emerging pathogens, whose incidence has increased importantly over the past two decades. *Prototheca wickerhamii* is a major human pathogen, responsible for at least 115 cases worldwide. Although the algae are receiving more attention nowadays, there is still a substantial knowledge gap regarding their biology, and pathogenicity in particular. Here we report, for the first time, the complete nuclear genome, organelle genomes, and transcriptome of the *P. wickerhamii* type strain ATCC 16529.

**Results:**

The assembled genome size was of 16.7 Mbp, making it the smallest and most compact genome sequenced so far among the protothecans. Key features of the genome included a high overall GC content (64.5%), a high number (6081) and proportion (45.9%) of protein-coding genes, and a low repetitive sequence content (2.2%). The vast majority (90.6%) of the predicted genes were confirmed with the corresponding transcripts upon RNA-sequencing analysis. Most (93.2%) of the genes had their putative function assigned when searched against the InterProScan database. A fourth (23.3%) of the genes were annotated with an enzymatic activity possibly associated with the adaptation to the human host environment. The *P. wickerhamii* genome encoded a wide array of possible virulence factors, including those already identified in two model opportunistic fungal pathogens, i.e. *Candida albicans* and *Trichophyton rubrum*, and thought to be involved in invasion of the host or elicitation of the adaptive stress response. Approximately 6% of the *P. wickerhamii* genes matched a Pathogen-Host Interaction Database entry and had a previously experimentally proven role in the disease development. Furthermore, genes coding for proteins (e.g. ATPase, malate dehydrogenase) hitherto considered as potential virulence factors of *Prototheca* spp. were demonstrated in the *P. wickerhamii* genome.

**Conclusions:**

Overall, this study is the first to describe the genetic make-up of *P. wickerhamii* and discovers proteins possibly involved in the development of protothecosis.

**Supplementary Information:**

The online version contains supplementary material available at 10.1186/s12864-021-07491-8.

## Background

The chlorophytan genus *Prototheca* contains aerobic, unicellular, colourless, yeast-like algae, able to cause disease in humans and other mammals, referred to as protothecosis. In fact, among all the Viridiplantae, only *Prototheca* and *Chlorella* microalgae possess a pathogenic potential for both humans and animals [1–4]. *Prototheca* spp. normally live as saprophytes and are environmentally ubiquitous, having been isolated from water, soil, slime flux of trees, raw and treated sewage, animal faeces, and food products [1, 5].

Since the first description of the *Prototheca* genus by Krüger in 1894 [6], its taxonomic position has been disputed for over a century, due to some apparent phenotypic similarities with yeasts. Currently, the *Prototheca* spp. are accepted to belong to the family Chlorellaceae of the order Chlorellales, in the class Trebouxiophyceae. Phylogenetically, their closest photosynthetic relative is *Auxenochlorella protothecoides* [7]. The *Prototheca* algae also share a close relationship with non-photosynthetic algae of the genus *Helicosporidium*, which are obligate parasites of arthropods, especially insects. Interestingly, the Helicosporidia seem to be basal to the *A. protothecoides* and *Prototheca* clades, implying that the loss of photosynthesis must have occurred at least twice in the evolution of heterotrophic Chlorellales [7].

The issue of molecular taxonomy of *Prototheca* spp. has been exhaustively addressed in a very recent work by Jagielski et al. [4]. Based on the partial *cytb* gene sequences, the genus was shown to accommodate 14 species. They all fell into two main lineages, i.e. cattle-associated (i.e. *Prototheca ciferrii*, formerly *Prototheca zopfii* gen. 1, *Prototheca blaschkeae*, and *Prototheca bovis*, formerly *Prototheca zopfii* gen. 2) and human-associated (i.e. *Prototheca wickerhamii*, *Prototheca cutis*, *Prototheca miyajii*) [4]. More recently, a new species of *Prototheca paracutis* has been described [8].

*Prototheca wickerhamii* is a major etiological agent of human protothecosis. The disease was first reported in 1964 in Sierra Leone [9], and since then, at least 211 new cases have been described in the literature [10]. Clinically, protothecosis manifests in three predominant forms, namely: cutaneous, olecranon bursitis, and disseminated or systemic disease. Protothecal infections are believed to develop through contact with potential sources (e.g. contaminated water), often following minor injuries or surgical interventions. Still, the exact portals of entry and mechanisms of pathogenesis in protothecosis remain obscure.

There is no standardized treatment protocol for protothecosis. Antifungal agents including the azoles (ketoconazole, itraconazole, fluconazole) and amphotericin B have been most commonly used, with the latter producing the best activity against *Prototheca* spp. [10].

The *Prototheca* algae and protothecosis have been much neglected areas of research. Studies on the genetic level are seriously lacking. Importantly, sequencing of the entire chromosomal DNA has so far been attempted in four species, i.e. *P. ciferrii*, *P. bovis*, *P. cutis*, and *Prototheca stagnora*, with the results released only in a draft format [11–13]. Although five reports have been published on mitochondrial and plastid genomes of *P. wickerhamii*, they all used the SAG 263–11 strain [7, 14–17], which in the light of the new *Prototheca* taxonomy represents not *P. wickerhamii*, but a completely different species, designated as *Prototheca xanthoriae* [4]. Only this year, has the first description of the organellar genomes of a true *P. wickerhamii* been published [18].

The objective of this study was to perform, for the first time, the genome-wide sequencing with thorough structural and functional analysis of the *P. wickerhamii* type strain, using a combination of PacBio and Illumina sequencing technologies. A subsequent transcriptome-proteome profiling was carried out to support the assembly completeness. This work also provides a first insight into protothecal pathogenesis, with several approaches used to select genes putatively involved in the virulence of *P. wickerhamii.*

For the comparative purposes, genomes of other *Prototheca* spp. and their closest relatives (*A. protothecoides* and *Helicosporidium* sp.) were included in the analysis. Furthermore, iconic fungal pathogens, including a yeast *Candida albicans* and a dermatophyte *Trichophyton rubrum* were used to search possible virulence factors. This was done due to some apparent phenotypic similarities (morphological and biochemical) shared between *P. wickerhamii* and fungi, particularly yeasts.

## Results and discussion

### General features of the *P. wickerhamii* nuclear genome

#### Nuclear genome assembly and quality assessment

Sequencing of the *P. wickerhamii* chromosome produced a total of 2,429,822,821 and 2,198,163,916 nucleotides and 8,239,274 and 286,004 reads for Illumina and PacBio, respectively. Those reads were further assembled into 21 contigs and as many scaffolds with an N50 length of 1,578,614 bp. The assembled sequencing data hence represented an average sequence depth of 150x, with the longest scaffold size of 2,447,261 bp. The high quality of the genome assembly was confirmed with the BUSCO analysis (Supplementary Figure S1). Furthermore, the RNA-mapping rate dataset supported the high assembly completeness. Among 10,963,602 read pairs from RNA-sequencing experiment, 90.64% uniquely mapped to the genome, while 0.94 and 0.01% mapped to multiple (> 1) or too many (> 10) loci, respectively. The vast majority (89.86%) of the mapped reads fell within predicted coding regions, suggesting that the total coding potential of the *Prototheca* organism was well-represented in the genome. Among predicted and annotated genes only 9.4% (573) did not have any overlaps with RNA-sequencing data.

#### Nuclear genome characteristics and gene prediction

The general features of the *P. wickerhamii* nuclear genome and its comparison to other analyzed genomes are shown in Table 1. The total assembly size was 16.7 Mbp. Gene structure of *P. wickerhamii*, reflected by average gene length, average number of introns/exons per gene, percentage of genes with introns, and mean intergenic length resembled *A. protothecoides* rather than *Helicosporidium* sp. (Table 1). All three algae shared similar GC-rich genomic composition, with higher GC content in exons compared to introns or intergenic regions (Table 1).

**Table 1:** Genome annotation statistics of *P. wickerhamii*, two closely related Chlorellales: *A. protothecoides* and *Helicosporidium sp.,* and two pathogenic fungi: *C. albicans* and *T. rubrum*. Data acquired from GFF files available at NCBI Genome (https://www.ncbi.nlm.nih.gov/genome)

Characteristic		*P. wickerhamii*	*A. protothecoides*	*Helicosporidium* sp.	*C. albicans*	*T. rubrum*
Sequencing	GenBank assembly accession (NCBI accession no. of assembly)	JADZLO010000000	GCA_000733215.1 (ASM73321v1)	GCA_000690575.1 (Helico_v1.0)	GCA_000182965.3 (ASM18296v3)	GCA_000151425.1 (ASM15142v1)
	Assembly length (Mb)	16.7	22.9	12.4	14.3	22.5
	Conting number	21	1386	5666	88	624
	N50 contig	1,578,614	35,091	3036	334,289	83,988
	Scaffold number	21	374	5666	8	36
	N50 scaffold	1,578,614	285,543	3036	2,231,883	2,156,965
	Genome coverage (Fold)	*ca.* 150x	145x	62x	700x	8.19x (7.49x Q > 20)
	Sequencing platform	PacBio; Illumina MiSeq 2 × 300	454 GS FLX Titanium; Illumina HiSeq 2000	Illumina HiSeq; Illumina GAIIx	Illumina GAIIx	Sanger ABI
GC content	GC content total (%)	64.5	63.5	61.7	33.5	48.3
	GC content exons (%)	68.7	68.1	66.5	35.1	51
	GC content introns (%) (between exons)	60.9	63	58.8	29.6	43
	GC content intergenic regions (%)	58.2	58.1	58.4	30.7	45.3
Protein coding genes	Number of genes	6081	7016	6033	6263	8804
	Average gene length (bp)	2135	2347	1031	1447	1572
	Average exon length (bp)	288	206	366	1336	454
	Average no of exons per gene	5.1	5.7	2.2	1.1	3.1
	Average intron length (bp)	162.8	247.2	170.2	146.1	85.4
	Average no of introns per gene	4.1	4.7	1.2	0.1	2.1
	Genes with introns (%)	97.4	88.7	56.3	6.8	81.7
	Mean intergenic lenght (bp)	1734.1	2184.4	1027	937.8	1108.1
	Coding sequence ratio (%)*	2.7	3.26	2.06	2.28	2.56
	Percentage coding	45.9	36.1	39.9	62.6	53.7
	Gene density (gene per Mb)	365.1	306.4	486.5	438.0	391.3
tRNA genes		64	71	29	126	100
Repetitive DNA in genome assembly (%)		2.25	1.98	1.23	4.6	1.89

*Coding sequence ratio = assembly length / number of genes * 1000

As for the other protothecal genomes, that of *P. wickerhamii* appeared to be the most compact, with structure highly similar to *P. cutis* [11, 12]. Since similar gene structure may suggest the evolutionary proximity between species [19], the data presented herein support close relatedness of *P. wickerhamii* and *P. cutis*.

The evolutionary proximity between *P. wickerhamii* and *P. cutis* was further supported with dendrogram analyses, based on 164 single copy genes shared among *Prototheca* species (Supplementary Figure S2).

A total of 6081 protein-encoding genes were predicted in *P. wickerhamii*, a number similar to *Helicosporidium* sp. and significantly lower than in *A. protothecoides* (Table 1). In terms of gene density, defined by the number of genes per Mbp, *P. wickerhamii* genome was similar to *A. protothecoides* (Table 1). The genome of *P. wickerhamii* was predicted to contain 960 less protein-coding genes than similar in size genome of *P. stagnora* [11, 12]. It thus seems that the genome size of *Prototheca* spp. is not associated with coding capacity. However, it cannot be excluded that the high number of genes in previously sequenced *Prototheca* species might have been overestimated as a consequence of potential fragmentation of genes into multiple individual contigs [20]. This miscalculation is very unlikely for our study, due to complete format of the genome.

Similar to *Helicosporidium* sp. [21] and *A. protothecoides, P. wickerhamii* genome encoded all tRNAs, except selenocysteine tRNA (Sec-tRNA) (Supplementary Table 1). In eukaryotes, the Sec insertion machinery is widespread in animals and green algae, while being absent in fungi and higher plants [22, 23].

#### Annotation of repetitive sequences

The percentage of repetitive sequences (i.e. interspersed low complexity regions and simple repeats - microsatellite regions) within the *P. wickerhamii* genome was comparable to that found in the genome of *A. protothecoides* and higher than in *Helicosporidium* sp. (Table 1). In all three algae, most of those elements were simple repeats (Supplementary Table 2). The interspersed repeats were extremely rare. Low number of interspersed repeats in small algal genomes is not surprising, since the genome size in eukaryotes is usually positively correlated with the repetitive sequences content [24].

Of note is that *P. wickerhamii* and *A. protothecoides*, in contrast to *Helicosporidium* sp., encoded Argonaute and Dicer proteins (Supplementary Figure S3), which are involved in silencing of the repetitive elements [25]. Those two proteins are found also in *Chlorella, Coccomyxa*, and *Chlamydomonas* genomes [26].

The majority of *P. wickerhamii* interspersed repeats were retroelements of which the long terminal repeat (LTR) elements Gypsy and Copia, predominated (Supplementary Table 2). Those two superfamilies are widely distributed among genomes of plants and fungi [27] including *Chlorella variabilis* [28] and *Candida albicans* [29].

Interestingly, an approximately 3-fold reduction of low complexity regions (LCRs) number in *P. wickerhamii* and *Helicosporidium* was observed, when compared to *A. protothecoides* (Supplementary Table 2). Low-complexity regions are tracts of single amino acids or short amino acid tandem repeats and may play a key role in the emergence of novel genes [30]. Thus, loss of low complexity regions in *P. wickerhamii* may reflect ongoing parasitic genome reduction.

### Plastid and mitochondrial genomes

The mitochondrial (mtDNA) and plastid (ptDNA) genomes of *P. wickerhamii* were comprehensively reported in our previous study [18]. Briefly, the circular mtDNA of *P. wickerhamii* was 53.8 kb in size, which is similar as in *Helicosporidium* sp. (49.3 kb), *A. protothecoides* (57.2 kb), and *P. xanthoriae* (55.3 kb), but not in other *Prototheca* spp., whose mtDNAs size was 38.3 kb (*P. ciferrii*) and 39.2 kB (*P. bovis*) (Supplementary Material 1; [18]). This could be explained by more complex intron structure in *P. wickerhamii, P. xanthoriae, A. protothecoides*, and *Helicosporidium* sp., when compared to *P. bovis* and *P. ciferrii*, and the presence of additional putative genes [18]. A typical set of 32 mitochondrial protein-coding genes was found in *P. wickerhamii* mtDNA and all but one were present among all the other microalgae studied (Supplementary Material 1; [18]). The exception was the *rpl10* gene, encoding for a ribosomal protein L10, found in *P. ciferrii* and *P. bovis*, yet not in *P. wickerhamii*. It has been shown, that during plant evolution, ribosomal protein genes, including *rpl10*, have been lost from the mitochondrion and transferred to the nucleus [31]. However, this rearrangement was not observed in *P. wickerhamii.*

The circular ptDNA of *P. wickerhamii* sized 48 kb, being larger than ptDNA of *P. ciferrii, P. bovis* (ca. 28.7 kB), and *Helicosporidium* sp. (37.4 kb), but smaller than that of photosynthetic *A. protothecoides* (84.6 kB) (Supplementary Material 1; [18]). The plastid genomes of *Prototheca* spp*.* and *Helicosporidium* sp. did not contain photosystem I and II proteins, cytochrome complex, and all genes for chlorophyll synthesis, when compared with *A. protothecoides*. In contrast to *Helicosporidium* sp. and other *Prototheca* spp., only *P. wickerhamii* and *P. xanthoriae* had all ribosomal proteins maintained. The differences in the gene content among *Prototheca* spp. may suggest that those algae discarded photosynthesis independently. Plastid genome-based phylogeny provided evidence for at least three independent losses (first in *P. xanthoriae*, the second in the ancestor of *P. wickerhamii* and *P. cutis,* and the third in *P. stagnora*, *P. bovis*, and *P. ciferrii*) [18].

### Photosynthesis-related genes

To further look at the *P. wickerhamii* genome reduction in terms of genes involved in photosynthesis, the inventory of proteins unique to plastid-containing organisms, GreenCut2 database was searched against the genome of *P. wickerhamii*. Overall, it encoded only 10 (13.5%) out of 74 photosynthesis-related, nuclear genes, predicted by the GreenCut2 database (Supplementary Material 2), whereas the photosynthetic *A. protothecoides* and non-photosynthetic *Helicosporidium* sp*.* encoded 54 (73%) and 8 (10.8%) of those genes, respectively. Eight out of 10 (80%) photosynthetic genes in *P. wickerhamii* were shared with *Helicosporidium* sp. Both *Helicosporidium* and *P. wickerhamii* did not encode proteins of light-harvesting antenna and photosystems I and II. Still, those two algae retained a component of the cytochrome b6/f complex (PetC) and PetF protein involved in the electron transport (Supplementary Material 2). Those data supported that those two non-photosynthetic trebouxiophytes had convergently lost a similar set of genes related to photosynthesis [11].

### Functional annotation of the nuclear genes

#### Prediction of domains, sites, repeats, and families among annotated genes

**Fig. 1:**
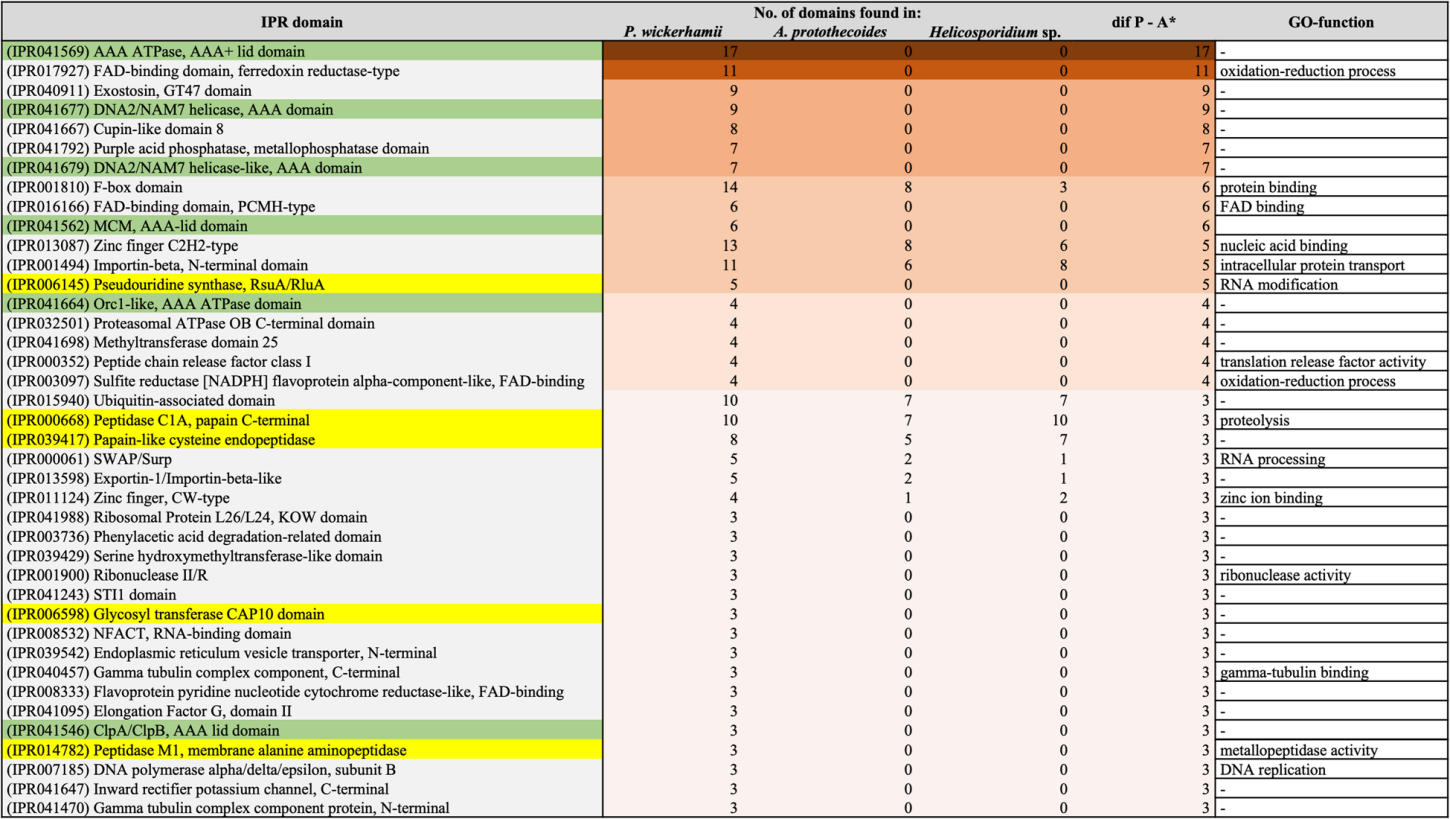
IPR domains most enriched in *P. wickerhamii* when compared to nonpathogenic *A. protothecoides****.*** Values are colored along a brown (high) to beige (low) color scale, with color scaling relative to the high and low values. Domains characteristic for AAA proteins are marked with green, and potentially involved in pathogenesis in yellow

The Interpro (IPR) resource provides functional analysis of the genes by predicting domains and important sites based on the signatures available in the database. To examine genes using this approach, IPR counts were compared between *P. wickerhamii*, *A. protothecoides*, and *Helicosporidium* sp. All these algae had similar percentage of genes in the genome mapped to each term among analyzed domains (Supplementary Material 3 and 4), sites (Supplementary Material 3 and 5), repeats (Supplementary Material 3 and 6), and families (Supplementary Material 3 and 7). Only 40 (1.9%) out of the total of 2065 Interpro domains were enriched in *P. wickerhamii* when compared to non-pathogenic *A. protothecoides* (with a difference set at ≥3 domains) (Fig. 1). Among those, domains with AAA motif were the most abundant (Fig. 1). The AAA proteins have been associated with various cellular processes including proteolysis, protein folding, membrane trafficking, cytoskeletal regulation, organelle biogenesis, DNA replication, and intracellular motility [32].

#### Functional analysis of the enzymes and prediction of proteases

Assigning enzymatic function to the genes was done using IPR signatures. Approximately a fourth (23.3%) of the genes were associated with enzymatic activity in *P. wickerhamii*, being comparable with *Helicospordium* sp. (25.5%), yet distant from *A. protothecoides,* where only 7.1% of genes had predicted enzyme activity (Supplementary Material 8).

**Fig. 2:**
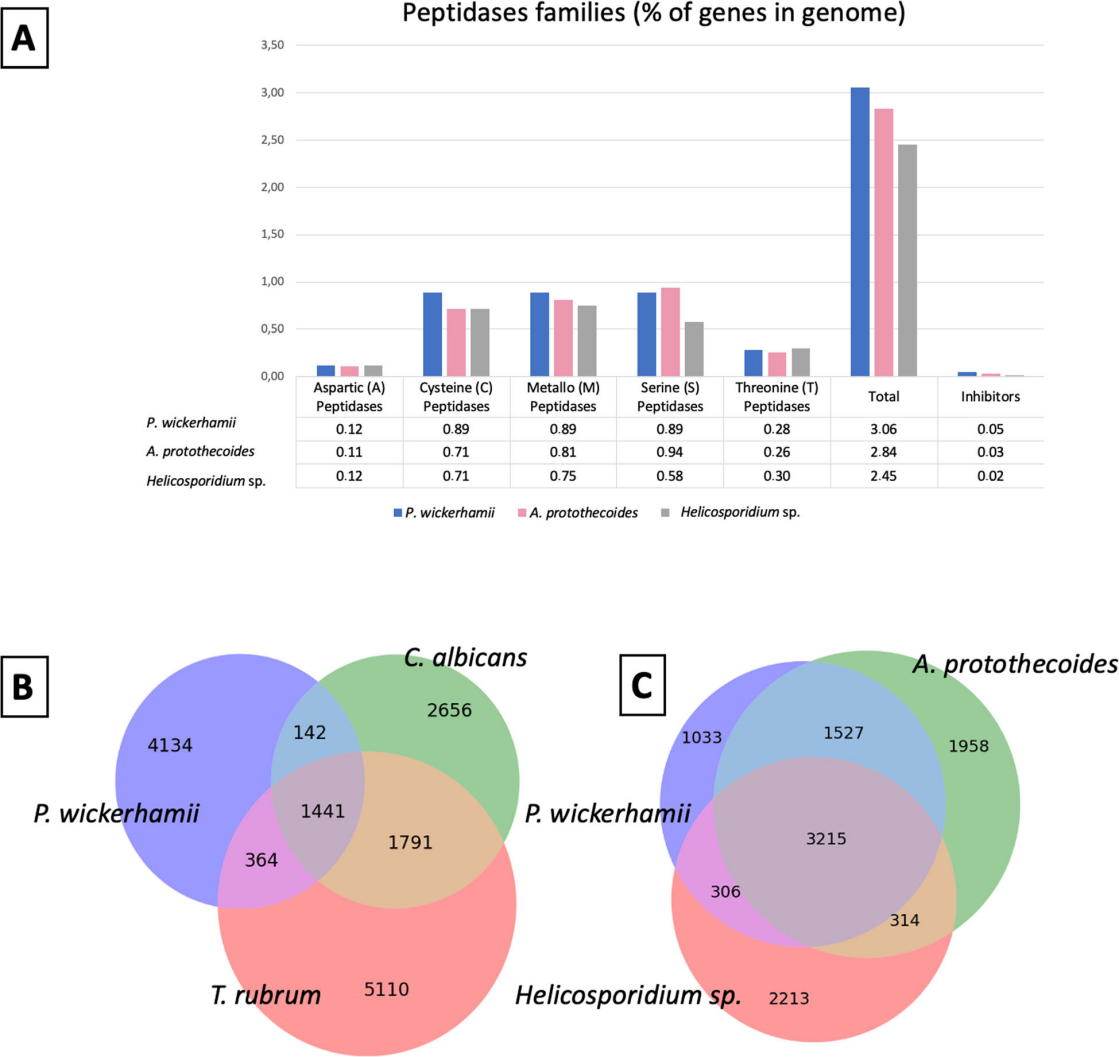
Comparative genomic analysis. **a** Peptidases found among all analyzed species using MEROPS database. **b** Reciprocal BLAST analysis of the predicted proteins among *P. wickerhamii* and two pathogenic fungi. The cut-off E-value is at <=1e-05. Each circle represents the relative fraction of genes represented in each of the categories. **c** Reciprocal BLAST analysis of the predicted proteins among *P. wickerhamii* and two closely related Chlorellales species. The cut-off E-value is at <=1e-05

Comparisons with the MEROPS peptidase database revealed that 3.1% of all genes in *P. wickerhamii* encoded peptidases (Supplementary Material 9), a number somewhat similar to *A. protothecoides* (2.8%) and *Helicospordium* sp. (2.4%) (Supplementary Material 10; Fig. 2a). Captivatingly, *P. wickerhamii* and *A. protothecoides* appeared to be particularly well equipped with serine peptidases when compared to *Helicospordium* sp. (Fig. 2a). Serine peptidases are extremely important in decomposing biomass, and have been frequently characterized in saprotrophs [33].

### Possible virulence factors

To disclose any possible virulence factors in *P. wickerhamii*, a four-step approach, combining (i) comparative genomics, (ii) cross-checking of virulence database (iii) searching for IPR domains overrepresented in *P. wickerhamii*, and (iv) searching for genes, whose proteins had previously been suggested to be associated with virulence in *Prototheca* spp.

#### Comparative genomics – *P. wickerhamii* versus fungal pathogens

A number of phenotypic features including morphology, antifungal drug susceptibility or opportunistic pathogenicity are shared between *P. wickerhamii* and certain fungi [1]. Thus, *P. wickerhamii* genome was compared with genomes of two model opportunistic fungal pathogens: *Candida albicans* and *Trichophyton rubrum*.

As many as 25.3% of *C. albicans* genes and 20.5% of *T. rubrum* genes were found in *P. wickerhamii* (Fig. 2b, Supplementary Material 11). Only 15 genes shared between *P. wickerhamii* and either *C. albicans* or *T. rubrum* had both predicted IPR domain and secretory signal (Table 2). Of those, 3 had glycoside hydrolases (GHs) domain of families 31 (IPR025887), 16 (IPR000757) and 20 (IPR015883). GHs cleave glycosidic bonds in polysaccharides and oligosaccharides and are important virulence factors in many species of bacteria [34] and plant-parasitizing fungi [35]. Notably, GH20 family represents putative virulence factors in oomycetes pathogenic to fish, crustaceans, and mosquitos, but are absent from phytopathogenic oomycetes *Phytophthora infestans* and *Phytophthora nicotianae* [36]. Furthermore, genes with saposin B and peptidase S8/S53 (subtilisin) domain were found, which had previously been described as virulence factors in pathogenic fungi, such as a thermodimorphic human pathogen *Histoplasma capsulatum* [37], *Pseudogymnoascus destructans*, a psychrophilic fungus that infects hibernating bats [38], and *Penicillium expansum*, a pathogen of apples and other fruit [39]. Approximately two-thirds (9/15; 60%) of the genes shared between *P. wickerhamii* and either *C. albicans* or *T. rubrum* with predicted IPR domain and secretory signal, had previously been characterized in either of the two fungi as related to pathogenicity (Table 2; Supplementary Material 12). Whereas APR1 and PEP1 genes of *C. albicans* and TERG_00899 of *T. rubrum* have been associated with penetration and invasion of the host [40–42], ROT2 and SKN1 of *C. albicans* have been linked to cell wall synthesis and mutants at these genes showed decreased in vitro virulence [43, 44]. Other genes found in *P. wickerhamii* were HEX1, GUT2, PNC1, and PDI1. The former allows for utilizing *N*-acetylglucosamine (GlcNAc) as a carbon source, which is an important virulence attribute of *C. albicans* [45]. Whereas, the other three are related to the adaptive stress response in *Candida* sp. [46–48].

**Table 2:** Secreted proteins shared between *P. wickerhamii* and either *C. albicans* or *T. rubrum*

No.	***C. albicans***		***T. rubrum***		Interpro (IPR) function
	Orth^**a**^	Gene	Orth^**a**^	Gene	
1.	1	CAALFM_CR00690CA; Uncharacterized protein	1	TERG_00664; Uncharacterized protein	Kinase
2.	0	–	1	TERG_04330; Uncharacterized protein	Folding of proteins in the endoplasmic reticulum
3.	0	–	1	TERG_01168; Uncharacterized protein	Involved in vacuolar sorting
4.	0	–	1	TERG_03290; Uncharacterized protein	Involved in nuclear export
5.	1	APR1, Proteinase A	1	TERG_06704; Vacuolar protease A	Involved in lipid metabolism/virulence
6.	1	GUT2; Glycerol-3-phosphate dehydrogenase	1	TERG_12172; Uncharacterized protein	Oxidoreductase activity
7.	1	PEP1; Sortilin	1	TERG_02098; Sortilin	Involved in vacuolar protein sorting
8.	1	PNC1; Nicotinamidase	1	TERG_06866; Uncharacterized protein	Catalytic activity/hydrolase
9.	1	CAALFM_CR00660WA; Uncharacterized protein	0	–	Proteolysis/virulence
10.	0	–	1	TERG_00899; Neutral ceramidase	Hydrolyses ceramide to generate sphingosine and fatty acid
11.	1	PDI1; Protein disulfide isomerase	1	TERG_04662; Protein disulfide-isomerase	Isomerase activity
12.	1	ROT2; Glucan 1\3-alpha-glucosidase	1	TERG_04559; Uncharacterized protein	Glycoside hydrolase
13.	1	SKN1; Skn1p	1	TERG_03740; Uncharacterized protein	Glycoside hydrolase
14.	1	HEX1; Beta-hexosaminidase	1	TERG_04775; Beta-hexosaminidase	Glycoside hydrolase
15.	1	NCR1; Sphingolipid transporter	1	TERG_02938; Uncharacterized protein	Involved in transport of cholesterol

^a^Existence of orthologs in *P. wickerhamii*; 0 – absent; 1 – present

#### Comparative genomics - *P. wickerhamii* unique genes

A total of 1033 genes were found exclusively in *P. wickerhamii* when compared with *A. protothecoides* and *Helicosporidium* sp. (Fig. 2c; Supplementary Material 13). Seventy-four (7.2%) contained known IPR domains, making their function predictable. Among genes with recognizable IPR domains were those demonstrated to be involved in response to hypoxia/phagocytosis (IPR001245), toxic substances (IPR004045), and cold-induced thermogenesis (IPR003736) [49–51]. This arsenal might be useful for *P. wickerhamii* to survive different environmental stresses that may confront it, while residing in the host or living saprophytically. Noteworthy, one *P. wickerhamii* unique protein contained LysM domain (IPR018392). This motif has been characterized in fungal plant pathogens, such as *Cladosporium fulvum* and *Magnaporthe oryzae* [52]. The LysM domain has also been enriched in several species of dermatophytes, including *T. rubrum*. It has been, however, unreported in *C. albicans*, *Malassezia globosa*, or *Pneumocystis jirovecii* [53]. The LysM effectors have been hypothesized to protect fungal cells against chitinases and other hydrolytic enzymes [52, 54].

Seventy-seven (7.4%) of the *P. wickerhamii* unique proteins contained predicted secretory signal, but only seven (0.7%) potentially secreted proteins had assigned IPR domain. Two genes contained domains potentially involved in pathogenesis, i.e. conferring proteolytic (PA domain; IPR003137) and hydrolytic (glycoside hydrolase, family 5; IPR001547) activity.

#### PHI-database cross-checking

**Fig. 3:**
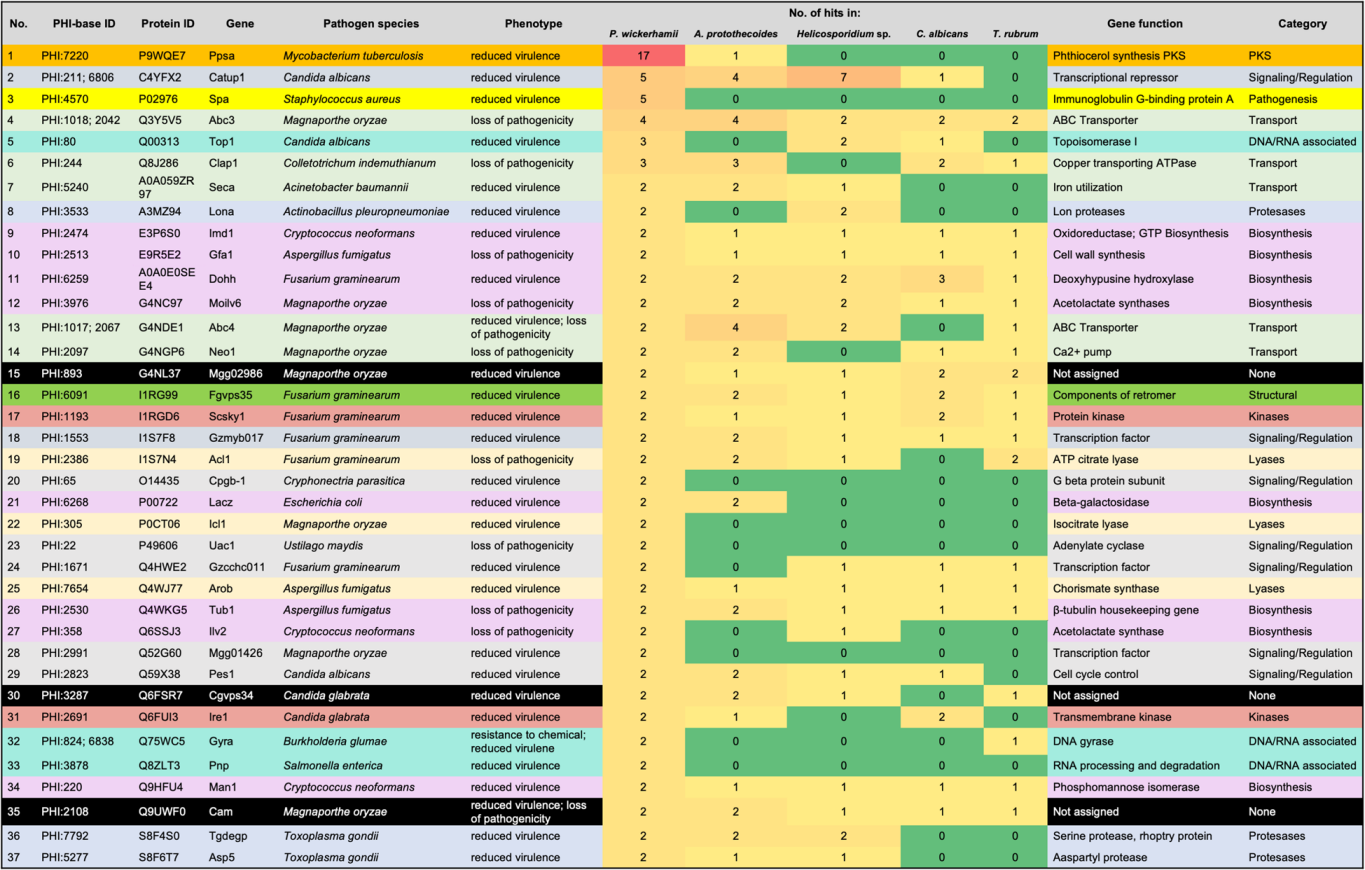
Highly represented (≥2) hits in PHI-base found in *P. wickerhamii* and their abundance in other analyzed genomes. Various row colors represent different functional categories. No. of hits are colored along a red (high) to yellow (low) color scale, with color scaling relative to the high and low values. When no of hits = 0, green color was used

Pathogen-Host Interaction Database (PHI database) was cross-checked to further identify genes potentially associated with pathogenicity in *P. wickerhamii.* Of the protothecal 593 genes matching a PHI-base entry, 373 (62.9%) had the annotation “reduced virulence” or “loss of pathogenicity”, indicating that their role in developing a disease has been experimentally proven (Supplementary Material 14). Among the highly represented (≥2) hits in the PHI-base (Fig. 3), two were characterized by the presence of ABC transporter domain (PHI:1018, PHI:2042 and PHI:1017, PHI:2067), thereby putatively involved in ATP-dependent export of organic anions or drugs from the cytoplasm. Multiple enzymes previously associated with fungal virulence such as oxidoreductases (PHI:2474), kinases (PHI:1193; PHI:2691), and lyases (PHI:305; PHI:2386; PHI:7654) were found. Whereas kinases were demonstrated to be expanded in dermatophytes [53], the importance of lyases for fungal virulence has been evidenced in *C. albicans* [55] and *T. rubrum* [56]. There were also as many as 17 hits to a polyketide synthase (PKS) *ppsA* (PHI:7220). PKSs are secondary metabolites, highly enriched in dermatophytes [53] and involved, for example, in the biosynthesis of melanin in *T. rubrum* during infection [57]. Furthermore, genes involved in host immune evasion in *Staphylococcus aureus* (PHI:4570) [58] and responsible for the yeast-to-hyphae transition, essential for *C. albicans* virulence (PHI:211; PHI:2823) were found. Finally, similar to *P. ciferrii* [13], *P. wickerhamii* encoded *hsp90* and *groEL*. Both these genes have been associated with the increased virulence in *C. albicans* [59] and a periodontopathic bacterium *Porphyromonas gingivalis* [60]*,* respectively.

#### Other possible virulence factors – IPR domains enriched in *P. wickerhamii*

Three peptidase domains, i.e. peptidase C1A, papain C-terminal (IPR000668), papain-like cysteine endopeptidase (IPR039417), and peptidase M1, membrane alanine aminopeptidase (IPR014782) were expanded in *P. wickerhamii* when compared to non-pathogenic *A. protothecoides* (Fig. 1). These domains have repeatedly been described as associated with pathogenesis. For instance, C1 peptidases, papain-like cysteine proteases (PLCPs), and alanine aminopeptidases play crucial roles in host/pathogen interactions in human pathogens, such as parasitic protozoa [61, 62] and various plant pathogens [63, 64]. Among other potential pathogenicity factors, proteins with glycosyl transferase CAP10 domain (IPR006598), known for capsule formation in *Cryptococcus neoformans* [65], were found in *P. wickerhamii*. Of particular note is the presence of pseudouridine synthase RsuA/RluA (IPR006145) domain in *P. wickerhamii* and its lack in *Helicosporidium* sp. and *A. protothecoides* (Fig. 1). In vitro transcribed RNAs containing various modified nucleotides, such as pseudouridine suppress the innate immune response in human cells through inactivation of pathogen’s RNA recognition by dendritic cells [66]. Captivatingly, none of the secreted endoproteases and exoproteases [67] (e.g. peptidase E, deuterolysin, fungalysin, aspergillopepsin I) from different human pathogenic fungi, including yeasts, molds, and dermatophytes summarized by Monod et al. [67] were present in *P. wickerhamii*. Accordingly, in *P. wickerhamii* other enzymes of proteolytic activity have to be involved in the early stages of infection of the host tissues.

#### Previously described *Prototheca* sp. virulence factors

Studies devoted to virulence factors in *Prototheca* spp. are very few. Irrgang et al. have identified several immunogenic proteins of *P. ciferrii*, *P. bovis*, and *P. blaschkeae* by assaying sera of *Prototheca*-infected animals by Western blotting [68, 69]. These finding, paralleled by a study of Murugaiyan et al. [70] have underlined the involvement of the housekeeping heat shock protein 70 (Hsp70) in the *P. bovis*-induced infections. Furthermore, among the immunodominant proteins identified by Irrgang et al. [68], elongation factor 1-α, glyceraldehyde-3-phosphate dehydrogenase, ATPase, and malate dehydrogenase exhibited differential expression profiles between pathogenic and non-pathogenic *Prototheca* species [63, 64, 70]. Genes coding for those 84 differentially expressed proteins, were searched against *P. wickerhamii* genome. All but 14 (70 or 83.3%) were found in the *P. wickerhamii* genome (Supplementary Material 15). Among them were ATPase, malate dehydrogenase, EF 1-α, and glyceraldehyde-3-phosphate dehydrogenase. Strikingly, Hsp70 protein (GenBank ID: E1Z7R4) was absent in *P. wickerhamii* (Supplementary Material 15). This may relate to the host preference of different *Prototheca* species. The Hsp70-harboring *P. bovis* affects mostly dairy cattle, while Hsp70-defficient *P. wickerhamii* is largely a human pathogen.

## Conclusions

To conclude, this paper provides a first insight into the genome of *P. wickerhamii* and discovers its general structural and functional features. The key findings can be summarized in five points. First, *P. wickerhamii* genome was the smallest among all protothecal genomes sequenced so far. Only an obligatorily pathogenic alga *Helicosporidium* sp. did display a more compact genome. Second, the structure of *P. wickerhamii* genome highly resembled that of closely related non-pathogenic green alga *A. protothecoides*. However, a high genome reduction was observed, as evidenced by the loss of low-complexity regions and photosynthesis-related genes. Third, *P. wickerhamii* showed a large battery of enzymes, possibly facilitating the adaptation of the alga to different ecological niches, including tissues of the parasitized host. Fourth, *P. wickerhamii* encoded numerous genes, which had previously been described as related to pathogenicity in fungi. Neither of these virulence factors, however, did represent the iconic fungal secreted endo- and exoproteases. Fifth, several new candidates for *P. wickerhamii*-specific virulence factors were described. Among these, were genes proven to affect the outcome of pathogen-host interactions reported in PHI-database, as well as *P. wickerhamii* unique genes with recognizable IPR domains involved in pathogenicity. Further experimental studies involving real-time PCR experiments and in vitro*/vivo* pathogenesis models are required to confirm role of these genes in *P. wickerhamii* virulence.

## Methods

### Strain

*P. wickerhamii* ATCC 16529 type strain, initially described by Tubaki & Soneda [71], purchased from American Type Culture Collection, and preserved in the Department of Medical Microbiology, Faculty of Biology, University of Warsaw collection, was used in this study. The strain was originally isolated from household plumbing in Peoria, IL (USA). It was stored in Viabank™ cryopreservative vial (Medical Wire & Equipment Co Ltd., Corsham, the UK), at − 70 °C and revived by streaking a loopful (10 μL) of the frozen culture on a Yeast Peptone Dextrose (YPD) (Difco, USA) agar plate and incubating at 37 °C aerobically for 72 h.

Cells of *P. wickerhamii* were then harvested from a single colony and grown in a 100 mL of YPD broth at 37 °C (200 rpm) until the absorbance at A600 reached approx. 5.0 (ca. 6.5 × 10^5^ CFU, 72 h).

### Genomic DNA extraction

Genomic DNA was isolated as described before [72] with a method based on three-pronged approach for whole cell lysis, i.e. mechanical (glass beads), enzymatic (Proteinase K) and surfactant-based (Triton-X100, SDS, and CTAB), disruption methods.

### Genome sequencing and assembly

The genome of *P. wickerhamii* was sequenced using a combination of Illumina (Illumina Inc., USA) and PacBio (Pacific Biosciences, USA) technologies.

The Illumina paired-end sequencing library construction was performed with 1 μg of post-nebulized DNA extract and the KAPA Library Preparation Kit reagents (KAPA Biosystems, USA), according to manufacturer’s instructions. The library was pooled and sequenced on a MiSeq platform using the 600-cycle MiSeq reagent Kit v.3 (Illumina, USA).

The PacBio libraries were constructed using approximately 20 μg of genomic DNA that was mechanically sheared to a size of 20 kb, using a Covaris gTube (Covaris, USA). Samples were then prepared by ligation of hairpin adaptors at both ends of the DNA fragment using the PacBio DNA template preparation kit 2.0 (Pacific Biosciences, USA). Libraries were purified using Agencourt AMPure beads (Beckman Coulter, USA) to remove fragments shorter than ca. 1.5 kb and size-selected using the BluePippin preparation system (Sage Science, USA) with a minimum cutoff of 7 kb. The sheared DNA and final library were characterized for size distribution using an Agilent Bioanalyzer 2100 (Agilent Technology, USA) along with a DNA12000 chip (Agilent Technology, USA). Single Molecule, Real-Time (SMRT) sequencing was carried out on the PacBio RS II using standard protocols (Pacific Biosciences, USA).

Once obtained, Illumina data were filtered by reads quality (> 20 qval) using FastaX [73], and the remaining sequencing adaptors were removed by Cutadapt [74]. PacBio long reads were filtered by length (> 1 kb) and quality (> 15 qval) using NanoFilt [75] and assembled de novo with wtdbg2 software (default parameters) [76]. Polishing long read assemblies with Illumina data was done with Pilon [77]. For further analyses the SeqMan (DNAStar, USA) and CLCBio Genomic Workbench pipeline for NGS (CLCBio, Denmark) were used.

### RNA sequencing and assembly

RNA samples for transcriptome analysis were extracted in duplicate from single culture condition at one time-point as described below. Cells of *P. wickerhamii* for RNA isolation were picked from a single colony and grown in a 50 mL of YPD broth at 37 °C with shaking (200 rpm) until the absorbance at A600 reached approx. 5.0 (ca. 6.5 × 10^5^ CFU, 72 h). Cells in the stationary phase were harvested by centrifugation (5000 rpm), and after decanting the medium, they were resuspended in StayRNA reagent (A&A Biotechnology, Poland), aliquoted into two equal portions and stored at − 70 °C until used.

RNA for transcriptome analysis was isolated from both cell portions using Total RNA kit (A&A Biotechnology, Poland), following manufacturer’s instructions, with an additional step of cell disruption. This was done by pulverization at 20 Hz for 15 min, using glass beads and TissueLyser II apparatus (Qiagen, Germany)*.* RNA degradation and genomic DNA contamination were monitored on 1% agarose gels. Samples were then treated with the RNase-free DNase (A&A Biotechnology, Poland) to remove any contaminating genomic DNA. RNA purity was checked using a NanoPhotometer® spectrophotometer (IMPLEN, USA) and the concentration was measured using a Qubit® RNA Assay Kit with a Qubit® 2.0 Flurometer (Thermo Fisher Scientific, USA). RNA integrity was assessed using the RNA 6000 Nano Assay Kit of the Bioanalyzer 2100 system (Agilent Technologies, USA). Finally, a total of 5 μg of RNA was used as input material for the libraries preparations. Sequencing libraries were generated using a Kapa Stranded mRNA Library Prep Kit for Illumina (KAPA Biosystems, USA) according to the producer’s protocol. The purified libraries were checked for quality and quantity using the Agilent 2100 Bionalyzer, Qubit®2.0 (Thermo Fisher Scientific, USA) and KAPA Library Quantification kit (Roche, Switzerland) and subsequently sequenced on a MiSeq instrument (Illumina, USA) with 2 × 75-bp paired-end reads.

The obtained reads were subjected to quality control and soft trimming using FastQC v0.11.6 and Trimmomatic v0.39. Adaptors sequences were removed alongside with low quality (> 20 qval) and short (> 15 bp) reads. Remaining sequences were used for transcriptome assembly by Trinity v2.1.1 with default settings for unguided assembly. Filtered reads were also mapped against created genome assembly using STAR v2.6.1a, followed by Cufflinks v2.2.1 [78] transcripts assembly. The fasta sequences of the transcripts was obtained from by bedtools v2.27.1. Downstream analysis and comparisons of collected transcripts, their isoforms and annotation were handled by custom bash and python scripts.

### Genome statistics

Genome statistics was calculated using GFF files obtained from NCBI Genome database (as referenced in Table 1; https://www.ncbi.nlm.nih.gov/genome; accessed 08.2018), and processed with BUSCO v3 [79] and QUAST [80]. If necessary, an in-house python script was implemented (available at https://github.com/henryk69/prototheca).

### Gene annotation

Genome completeness was assessed using BUSCO v3 with eukaryota_odb9 database [79]. Scaffold sequences were masked with RepeatMasker v4.0.8 using the Viridiplantae section [81]. Gene prediction and annotation was performed using the MAKER v2 annotation pipeline [82]. For *ab initio* gene prediction, a combination of predictions from GeneMark-ES v4.10 [83] and AUGUSTUS v3.2.3 [84] trained with BRAKER v2.0 [85] using genome and data derived from RNA-sequencing was used. To generate protein-based evidence Expressed Sequence Tags (ESTs), a database was prepared. A total of 34,771 sequences from the Chlorellales family i.e. *Helicosporidium* sp., *A. protothecoides*, *Parachlorella kessleri*, *Chlorella vulgaris*, *Chlorella variablilis* and *Chlorella pyrenoidosa*, and 6805 sequences deposited for *P. wickerhamii* (derived from the sequencing of plastid and mitochondrial genome) available at NCBI database were included. EST, RNA-sequencing data and ab initio gene predictors were all used with the MAKER pipeline to iteratively obtain the final gene annotations.

tRNAscan v1.4 [86] was used to predict tRNA genes.

RepeatMasker 4.0 with RepBase was used to identify and mask interspersed low-complexity regions as well as simple repeats (micro-satellites) in the DNA [87]. The Viridiplantae dataset was used to define transposable elements present in the analyzed Chlorellales genomes.

All sequence similarity was assessed using BLAST v2.2.24 [88]) with E-value cutoff 1e-10. Sequence alignments were prepared using Exonerate [89].

GeneOntology annotations were obtained from Blast2GO [90]. Blast2GO was also used for InterProScan [91] domain annotations. GreenCut [92], PHI-base [93] and Merops [94] databases were searched with a reciprocal procedure using BLASTP with E-value 1e-10. Dicer and Argonaute *viridiplantae* proteins were obtained from UniprotKB [95] database.

Comparative genomics assessment was done using a reciprocal BLASTP procedure used in [96] to identify putative orthologous proteins between all three *Chlorellales* and two fungi genomes based on one-to-one reciprocal best BLASTP hits. All reciprocal BLASTP searches between were performed with an E-value cutoff of 1e− 10 and identified protein pairs which were reciprocally one another’s top BLASTP hit and that occurred once and only once in each proteome query. All obtained annotation and protein similarity results were assessed with in-house built python scripts using standard pandas, numpy, matplotlib, seaborn, and other libraries (available at https://github.com/henryk69/prototheca).

Genes coding for 84 differentially expressed proteins between pathogenic and non-pathogenic *Prototheca* species [63, 64, 70] were used as identifiers for UniprotKB database. All 84 proteins were obtained in their cannonical form. Reciprocal BLAST procedure was done with E-value threshold set to e-10 and at least 40% sequence similarity to ensure genuine resemblance between the *P. wickerhamii* proteome and the 84 proteins.

### Phylogenetic analysis

Phylogenetic tree was prepared using single copy conserved genes retrieved by BUSCO with eucaryotic_odb9 gene dataset. A total of 164 universal single copy genes were shared among all 5 *Prototheca* species, with available WGS data ([11–13]; this manuscript).

Protein sequences (products of the 164 single copy genes) were aligned with MAFFT (v7.475) [97] with default parameters and trimmed with trimAl (v. 1.2rev59) [98] with the strictplus option enabled. To generate the phylogenetic tree from the concatenated alignment RAxML (v8.2.12) [99] was used with options: rapid bootstrap analysis (−f a), 100 boostrap samples (−N 100) and GAMMA model of rate heterogeneity with automatically chosen best protein substitution model (−m PROTGAMMAAUTO). EvolView [100] was used for tree visualization.

## Supplementary Information

**Additional file 1: Supplementary Figure S1.** Completeness of the analyzed genomes assessed with BUSCO. The red, yellow, dark blue and light blue bar chart shows the % of missing (M), fragmented (F), complete (C) and duplicated (D), complete (C) and single copy (S) genes in the assemblies, respectively.

**Additional file 2: Supplementary Figure S2.** Maximum likelihood (ML) phylogenetic tree based on the 164 universal single copy genes shared among *Prototheca* species. The phylogenetic tree shows, that *P. wickerhamii* is closely related to *P. cutis* and more distantly to *P. stagnora*. *P. bovis* and *P. ciferrii* seem evolutionary closer to each other than to *P. wickerhamii*. The presented herein architecture is the same for all 100 bootstrapped trees.

**Additional file 3 Supplementary Figure S3**. Dicer and Argonuate proteins found within *P. wickerhamii* (A) and *A. protothecoides* (B) genomes. In *P. wickerhamii* Dicer protein resembles the DCL2 from *A. thaliana* (e-value = 5.75e-60; sequence similarity: > 56%) whereas the Argonaute protein - AGO_10 from *A. thaliana* (e-value = 8e-103, > 45% similarity).

**Additional file 4: Supplementary Table 1.** List of tRNA gene sequences found in the *P. wickerhamii* and *A. protothecoides*.

**Additional file 5: Supplementary Table 2.** Repetitive DNA elements found in the *P. wickerhamii*, two closely related Chlorellales: *A. protothecoides* and *Helicosporidium sp.,* and two pathogenic fungi: *C. albicans* and *T. rubrum*.

**Additional file 6: Supplementary Materials.**

## Data Availability

This Whole Genome Shotgun project of *P. wickerhamii* has been deposited at DDBJ/ENA/GenBank under the accession JADZLO000000000. The version described in this paper is version JADZLO010000000. All in-house python scripts are available at https://github.com/henryk69/prototheca. Furthermore, the datasets supporting the conclusions of this article are available as Supplementary Tables 1&2 and Supplementary Material.
