# Supplementary figures and images for: A first insight into the genome of Prototheca wickerhamii, a major causative agent of human protothecosis
Source: BMC Genomics. 2021 Mar 9;22:168. doi: 10.1186/s12864-021-07491-8 (PMC7941945; doi:10.1186/s12864-021-07491-8)

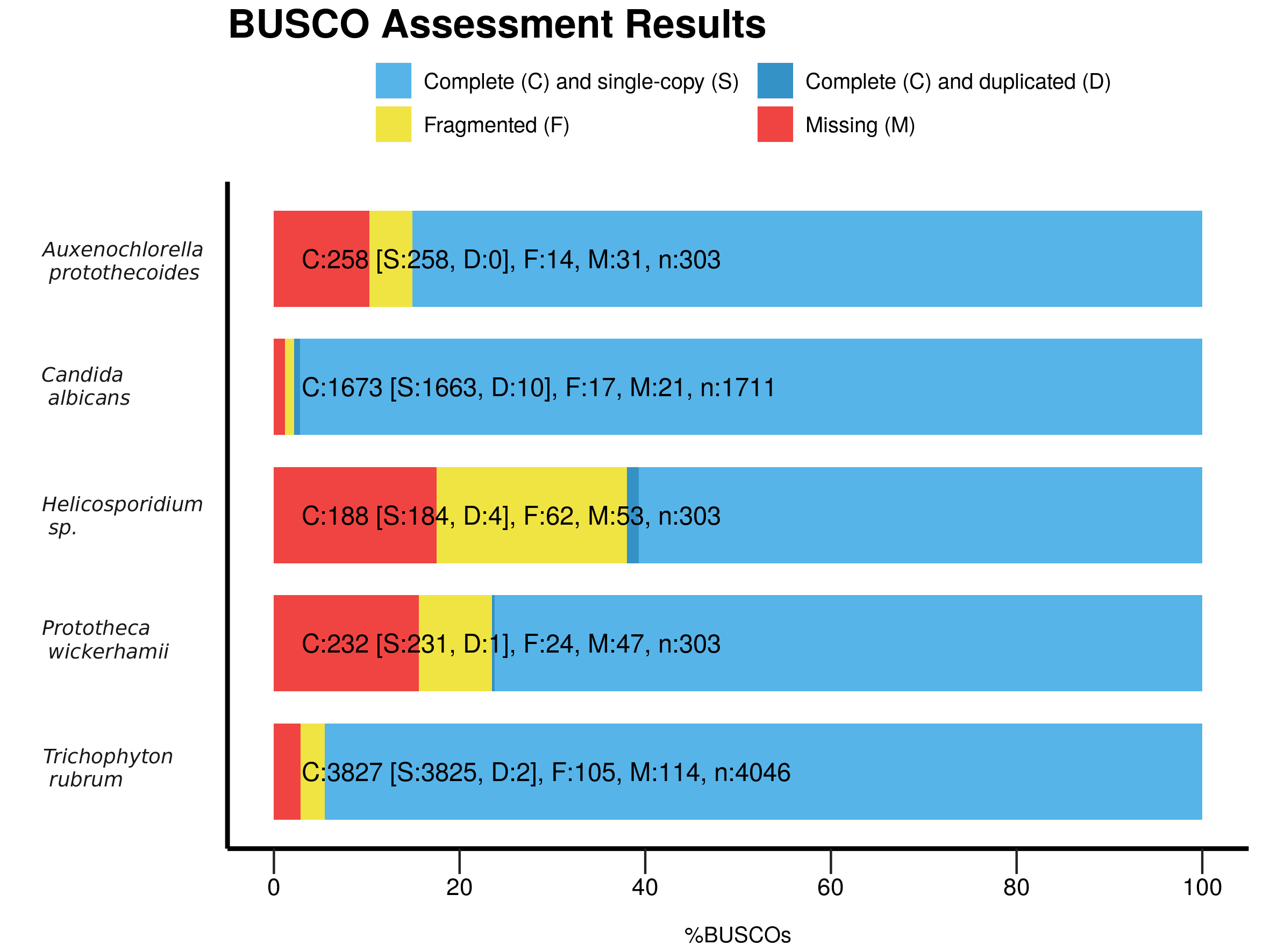

Supplement: Supplementary file 1 — Additional file 1: Supplementary Figure S1. Completeness of the analyzed genomes assessed with BUSCO. The red, yellow, dark blue and light blue bar chart shows the % of missing (M), fragmented (F), complete (C) and duplicated (D), complete (C) and single copy (S) genes in the assemblies, respectively. [file 12864_2021_7491_MOESM1_ESM.png]

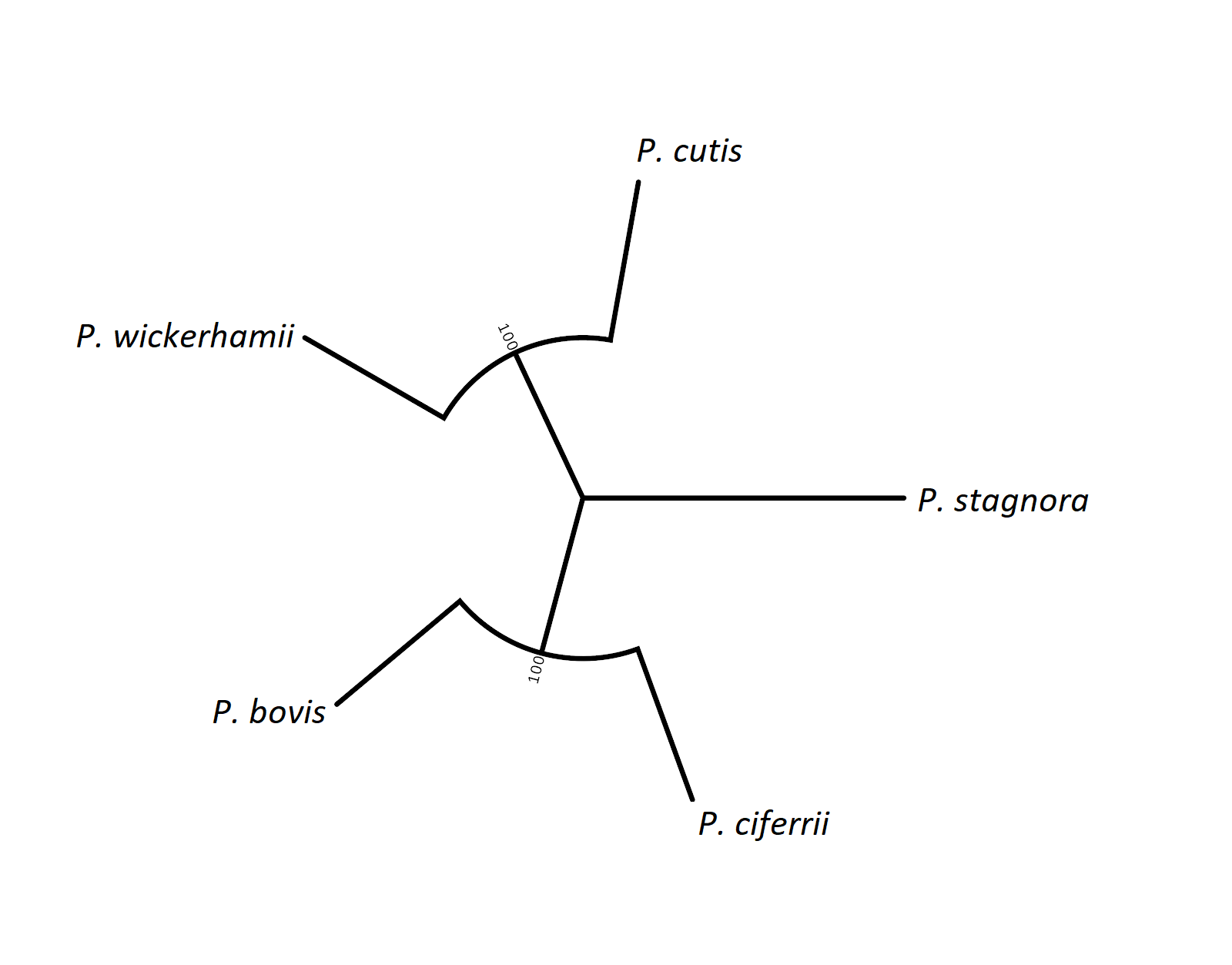

Supplement: Supplementary file 2 — Additional file 2: Supplementary Figure S2. Maximum likelihood (ML) phylogenetic tree based on the 164 universal single copy genes shared among Prototheca species. The phylogenetic tree shows, that P. wickerhamii is closely related to P. cutis and more distantly to P. stagnora. P. bovis and P. ciferrii seem evolutionary closer to each other than to P. wickerhamii. The presented herein architecture is the same for all 100 bootstrapped trees. [file 12864_2021_7491_MOESM2_ESM.png]

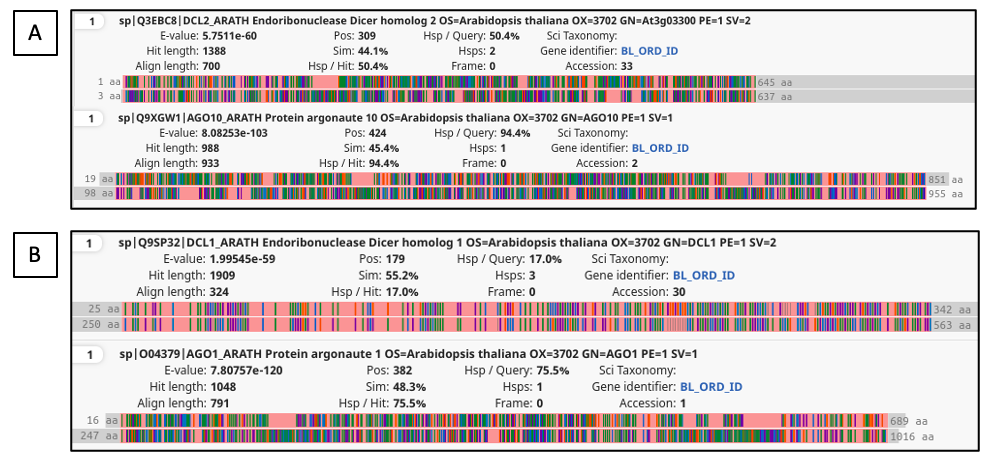

Supplement: Supplementary file 3 — Additional file 3 Supplementary Figure S3. Dicer and Argonuate proteins found within P. wickerhamii (A) and A. protothecoides (B) genomes. In P. wickerhamii Dicer protein resembles the DCL2 from A. thaliana (e-value = 5.75e-60; sequence similarity: > 56%) whereas the Argonaute protein - AGO_10 from A. thaliana (e-value = 8e-103, > 45% similarity). [file 12864_2021_7491_MOESM3_ESM.png]
