# Supplementary material for: A first insight into the genome of Prototheca wickerhamii, a major causative agent of human protothecosis
Source: BMC Genomics. 2021 Mar 9;22:168. doi: 10.1186/s12864-021-07491-8 (PMC7941945; doi:10.1186/s12864-021-07491-8)
Supplement: Supplementary file 4 — Additional file 4: Supplementary Table 1. List of tRNA gene sequences found in the P. wickerhamii and A. protothecoides. [file 12864_2021_7491_MOESM4_ESM.docx]

**Supplementary Table 1.** List of tRNA gene sequences found in the P. wickerhamii and *A. protothecoides*.

| **No.** | **Aminoacid** | **Codon** | **No. of tRNAs found in:** | |
| --- | --- | --- | --- | --- |
|  |  |  | ***P. wickerhamii*** | ***A. protothecoides*** |
| 1. | Ala | TGC | 1 | 1 |
| 2. | Arg | ACG | 4 | 4 |
|  |  | CCT | 1 | 1 |
|  |  | TCT | 2 | 2 |
|  |  | CCG | 1 | 1 |
| 3. | Asn | GTT | 2 | 2 |
| 4. | Asp | GTC | 5 | 5 |
| 5. | Cys | GCA | 2 | 2 |
| 6. | Gln | TTG | 2 | 2 |
| 7. | Glu | TTC | 2 | 2 |
| 8. | Gly | GCC | 2 | 2 |
|  |  | TCC | 2 | 1 |
| 9. | His | GTG | 2 | 2 |
| 10. | Ile | GAT | 1 | 2 |
| 11. | Leu | CAA | 1 | 2 |
|  |  | CAG | 2 | 3 |
|  |  | TAG | 3 | 3 |
|  |  | TAA | 1 | 1 |
| 12. | Lys | TTT | 1 | 1 |
| 13. | Met | CAT | 7 | 7 |
| 14. | Phe | GAA | 4 | 4 |
| 15. | Pro | AGG | 2 | 2 |
|  |  | CGG | 1 | 1 |
|  |  | TGG | 2 | 2 |
| 16. | Ser | TGA | 2 | 2 |
|  |  | GCT | 1 | 2 |
|  |  | GGA | 0 | 1 |
| 17. | Thr | TGT | 2 | 2 |
|  |  | GGT | 0 | 1 |
| 18. | Trp | CCA | 1 | 1 |
| 19. | Tyr | GTA | 2 | 2 |
| 20. | Val | TAC | 1 | 2 |
| 21. | Other | Pseudo_GCT | 1 | 0 |
|  |  | Pseudo_CAA | 1 | 0 |
|  |  | Pseudo_GAG | 0 | 1 |
|  |  | Pseudo_TCC | 0 | 1 |
|  |  | Pseudo_NNN | 0 | 1 |
| **TOTAL** | | | **64** | **71** |
