# Supplementary material for: A first insight into the genome of Prototheca wickerhamii, a major causative agent of human protothecosis
Source: BMC Genomics. 2021 Mar 9;22:168. doi: 10.1186/s12864-021-07491-8 (PMC7941945; doi:10.1186/s12864-021-07491-8)
Supplement: Supplementary file 5 — Additional file 5: Supplementary Table 2. Repetitive DNA elements found in the P. wickerhamii, two closely related Chlorellales: A. protothecoides and Helicosporidium sp., and two pathogenic fungi: C. albicans and T. rubrum. [file 12864_2021_7491_MOESM5_ESM.docx]

**Supplementary Table 2.** Repetitive DNA elements found in the P. wickerhamii, two closely related Chlorellales: A. protothecoides and *Helicosporidium* sp.*,* and two pathogenic fungi: C. albicans and T. rubrum.

|  |  | **Family** | | ***P. wickerhamii*** | | ***A. protothecoides*** | | ***Helicosporidium* sp.** | | ***C. albicans*** | | ***T. rubrum*** | |
| --- | --- | --- | --- | --- | --- | --- | --- | --- | --- | --- | --- | --- | --- |
|  |  |  |  | Number of elements  (% of sequence) | Occupied length [bp] | Number of elements  (% of sequence) | Occupied length [bp] | Number of elements  (% of sequence) | Occupied length [bp] | Number of elements  (% of sequence) | Occupied length [bp] | Number of elements  (% of sequence) | Occupied length [bp] |
| **Interspreads repeats** | **Retroelements** | **Total** | | **52 (0.05)** | 8607 | **54 (0.04%)** | 8840 | **66 (0.06%)** | **7581** | **219 (0.48%)** | **68305** | **128 (0.09%)** | **10818** |
|  |  | SINEs | | 1 (0.00) | 74 | 0 (0.00) | 0 | 0 (0.00) | 0 | 0 | 0 | 0 (0.00) | 0 |
|  |  | LINEs: | | 5 (0.00) | 475 | 9 (0.01) | 1404 | 6 (0.00) | 430 | 10 (0.07) | 9469 | 59 (0.04) | 9358 |
|  |  |  | CRE/SLACS | 0 (0.00) | 0 | 0 (0.00) | 0 | 0 (0.00) | 0 | 0 (0.00) | 0 | 1 (0.00) | 35 |
|  |  |  | RTE/Bov-B | 1 (0.00) | 163 | 2 (0.00) | 477 | 0 (0.00) | 0 | 0 (0.00) | 0 | 0 (0.00) | 0 |
|  |  |  | L1/CIN4 | 4 (0.00) | 312 | 5 (0.00) | 787 | 6 (0.00) | 430 | 0 (0.00) | 0 | 0 (0.00) | 0 |
|  |  | LTR: | | 46 (0.05) | 8058 | 45 (0.03) | 7436 | 60 (0.00) | 7151 | 209 (0.41) | 58836 | 69 (0.05) | 10460 |
|  |  |  | Gypsy/DIRS1 | 29 (0.04) | 6061 | 34 (0.03) | 5796 | 34 (0.00) | 5466 | 59 (0.09) | 13021 | 44 (0.04) | 8455 |
|  |  |  | Ty1/Copia | 14 (0.01) | 1534 | 9 (0.01) | 1338 | 24 (0.00) | 1446 | 99 (0.21) | 29944 | 25 (0.01) | 2005 |
|  | **DNA transpozons** | **Total** | | **34 (0.02)** | **3143** | **28 (0.01)** | **2426** | **30 (0.03)** | **3672** | **29 (0.06)** | **8280** | **46 (0.04)** | **9693** |
|  |  |  | hobo-Activator | 7 (0.00) | 597 | 7 (0.00) | 493 | 8 (0.01) | 787 | 0 (0.00) | 0 | 1 (0.00) | 40 |
|  |  |  | Tc1-IS630-Pogo | 1 (0.00) | 66 | 0 (0.00) | 0 | 0 (0.00) | 0 | 8 (0.05) | 6884 | 14 (0.03) | 5988 |
|  |  |  | Other* | 1 (0.00) | 217 | 1 (0.00) | 54 | 0 (0.00) | 0 | 1 (0.00) | 40 | 0 (0.00) | 0 |
|  |  |  | PiggyBac | 0 (0.00) | 0 | 0 (0.00) | 0 | 0 (0.00) | 0 | 0 (0.00) | 0 | 1 (0.00) | 182 |
|  | **Unclassified** | | | **7** (0.01) | **936** | **6** (0.00) | **651** | **6 (0.01)** | **692** | **10 (0.17)** | **24205** | **1** (0.00) | **61** |
|  | **Total** | | | **93 (0.08)** | **12686** | **88 (0.05)** | **11917** | **102 (0.1)** | **11945** | **258 (0.71)** | **100790** | **175 (0.13)** | **29572** |
| Small RNA | | | | 11 (0.02) | 3719 | 9 (0.03) | 6575 | 4 (0.02) | 2097 | 3 (0.04) | 5183 | 23 (0.01) | 2564 |
| Satellites | | | | 17 (0.01) | 2115 | 16 (0.01) | 1654 | 12 (0.01) | 1276 | 3 (0.00) | 180 | 3 (0.00) | 145 |
| Simple repeats | | | | 6196 (1.97) | 329171 | 7797 (1.59) | 365421 | 2575 (0.93) | 115284 | 11709 (3.27) | 466868 | 8117 (1.44) | 32433 |
| Low complexity | | | | 503 (0.17) | 28141 | 1431 (0.3) | 68091 | 442 (0.18) | 22215 | 1720 (0.59) | 84486 | 1328 (0.31) | 70131 |

*Other DNA transpozon families i.e.: Mirage, P-element or Transib.
